# The role of polymorphism in various potential genes on polycystic ovary syndrome susceptibility and pathogenesis

**DOI:** 10.1186/s13048-021-00879-w

**Published:** 2021-09-26

**Authors:** Hiral Chaudhary, Jalpa Patel, Nayan K. Jain, Rushikesh Joshi

**Affiliations:** 1 Department of Biochemistry and Forensic Science, University School of Sciences, Gujarat University, Ahmedabad, Gujarat 380009 India; 2 Department of Life Science, University School of Sciences, Gujarat University, Ahmedabad, Gujarat 380009 India

**Keywords:** Polycystic ovary syndrome, Hyperandrogenism, Ovarian steroidogenesis, Gonadotropins, Candidate genes, Polymorphism

## Abstract

Polycystic ovary syndrome (PCOS) is the most common endocrinopathies affecting the early reproductive age in women, whose pathophysiology perplexes many researchers till today. This syndrome is classically categorized by hyperandrogenism and/or hyperandrogenemia, menstrual and ovulatory dysfunction, bulky multi follicular ovaries on Ultrasonography (USG), and metabolic abnormalities such as hyperinsulinemia, dyslipidemia, obesity. The etiopathogenesis of PCOS is not fully elucidated, but it seems that the hypothalamus-pituitary-ovarian axis, ovarian, and/or adrenal androgen secretion may contribute to developing the syndrome. Infertility and poor reproductive health in women’s lives are highly associated with elevated levels of androgens. Studies with ovarian theca cells taken from PCOS women have demonstrated increased androgen production due to augmented ovarian steroidogenesis attributed to mainly altered expression of critical enzymes (Cytochrome P450 enzymes: CYP17, CYP21, CYP19, CYP11A) in the steroid hormone biosynthesis pathway. Despite the heterogeneity of PCOS, candidate gene studies are the widely used technique to delineate the genetic variants and analyze for the correlation of androgen biosynthesis pathway and those affecting the secretion or action of insulin with PCOS etiology. Linkage and association studies have predicted the relationship between genetic variants and PCOS risk among families or populations. Several genes have been proposed as playing a role in the etiopathogenesis of PCOS, and the presence of mutations and/or polymorphisms has been discovered, which suggests that PCOS has a vital heritable component. The following review summarizes the influence of polymorphisms in crucial genes of the steroidogenesis pathway leading to intraovarian hyperandrogenism which can result in PCOS.

## Introduction

Polycystic ovary syndrome (PCOS) is the most common endocrinopathies, first reported in 1935 by Stein I. F and Leventhal M. L [1]. WHO’s estimated ratio of PCOS affecting women of reproductive age group worldwide is 116 million (3.6%) [2]. Globally, the prevalence of PCOS is varying from 2.2% to as high as 26%. Based on the 1990 US National Institutes of Health (NIH) diagnostic criteria, the prevalence rate from the United States, Europe, Asia, and Australia is between 5 to 9% and approximately between 4 to 21% when Rotterdam 2003 criteria is applied in clinically evident PCOS women of reproductive age [3]. In India, the prevalence estimate is 10% and yet no clear statistical data is available [4].

PCOS is majorly characterized by hyperandrogenism and/or hyperandrogenemia, menstrual and ovulatory dysfunction manifested as oligomenorrhea, amenorrhea or chronic anovulation, and polycystic ovarian morphology (PCOM: an excessive number of preantral follicles in ovaries) [5]. Clinical Hyperandrogenemia leads to excessive terminal hair growth on the face or body suggesting masculine features known as hirsutism and leads to cosmetic consequences such as acne and alopecia (male pattern baldness). In contrast, biochemical hyperandrogenism results in excessive production of androgens and insulin resistance [6]. It is also associated with metabolic risk factors including hyperinsulinemia, Type II Diabetes mellitus, hypertension, dyslipidemia, and cardiovascular disorders [7].

An alteration observed in the steroid biosynthesis pathway increases the androgen levels in PCOS women [8]. Most of the enzymes involved in the biosynthesis of the adrenal steroid hormones and the gonadal steroid hormones fall into two major classes of proteins: the cytochrome P450 heme-containing proteins and the hydroxysteroid dehydrogenases. The P450 enzymes involved in steroid hormone biosynthesis are membrane-bound proteins associated with the mitochondrial membranes CYP11A, CYP11B1, and CYP11B2, or the endoplasmic reticulum (microsomal) CYP17, CYP19, and CYP21. Studies have shown that hyperandrogenism, luteinizing hormone (LH) hypersecretion, hyperinsulinemia are majorly associated with the pathophysiology of PCOS [9]. Hyperandrogenism is prominently observed in the ovary of PCOS women, which leads to intense ovarian steroidogenesis [10]. For ages, ovarian function is affected by androgens which are often associated with infertility. Androgen excess is the main factor promoting anovulation and follicular arrest, suggesting decreased oocyte development and maturation [11]. Several genes have been proposed as playing a role in the etiopathogenesis of PCOS, and the presence of mutations and/or polymorphisms has been discovered. However, their exact role is still not clear [12]. The central genes explored in the steroidogenesis pathway and gonadotropin action and regulation in developing PCOS are explained in this review article.

## Etio-pathogenesis

The term polycystic ovary syndrome was coined after Stein and Leventhal studied ovarian morphology and histology and numerous clinical findings that verified the existence of polycystic ovaries in women [13]. The National Institutes of Health (NIH) Conference suggested in 1990 when the diagnostic criteria for PCOS were first introduced that both hyperandrogenism and chronic anovulation be always present [14]. Later, in 2003, the ESRHE/ASRM Rotterdam criteria specified PCOS by requiring at least two of three characteristics of oligo-ovulation/anovulation, hyperandrogenism, and polycystic ovaries on USG to be present [15].. In 2006, the Androgen Excess Society (AES) proposed an amendment, in which oligo-ovulation/anovulation or polycystic ovaries on ultrasonography should accompany a clinical or biochemical diagnosis of hyperandrogenism [16].

**Fig. 1:**
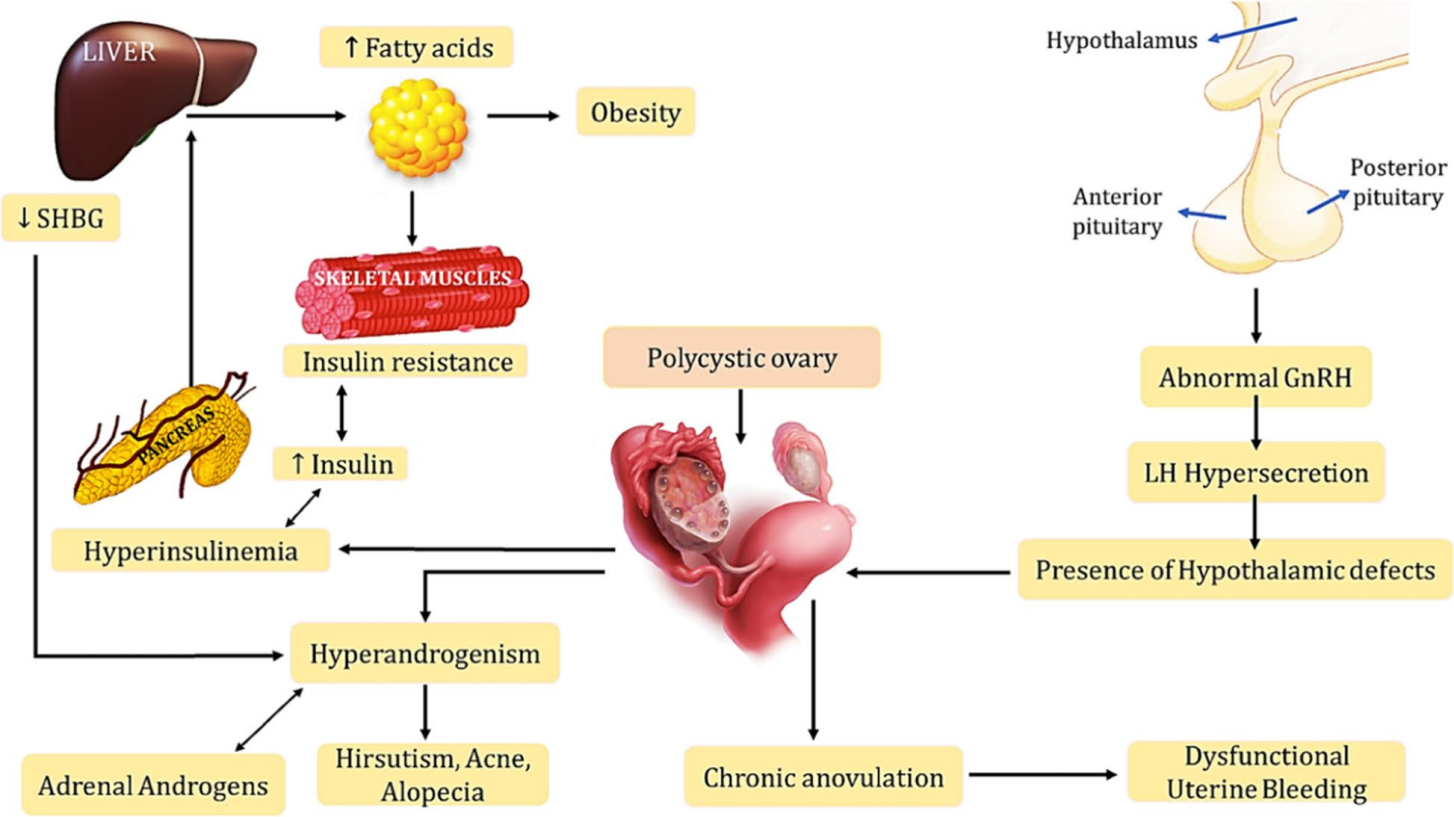
Implications of Polycystic ovary syndrome in women’s life. The pulsatile release of gonadotropin-releasing hormone (GnRH) from the hypothalamus often leads to hyperandrogenism and polycystic ovaries through hypersecretion of luteinizing hormone indicating hypothalamic defects. Low levels of sex hormone-binding globulin (SHBG) and adrenal androgens might also result in hyperandrogenism which is mostly observed in PCOS. Abbreviations: LH, luteinizing hormone; GnRH, gonadotropin-releasing hormone; PCOS, polycystic ovary syndrome, SHBG; sex hormone-binding globulin

PCOS being a multigenic trait, described in Fig. 1: Implications of Polycystic ovary syndrome in women’s lives; many pathways may be involved in its etiology. Researchers are studying PCOS for ages and generated many hypotheses about the PCOS development and its characteristics features, but the etiology behind the syndrome is still unclear. The pathogenesis of PCOS is associated primarily with theca cell defects along with neuroendocrine dysfunction of the hypothalamic-pituitary-ovarian axis resulting in hyperandrogenism [17]. In normal conditions, the hypothalamus signals the pituitary gland to release Gonadotropin-releasing hormone (GnRH), which further stimulates the normal signaling pathway for releasing Luteinizing hormone (LH) and Follicle-stimulating hormone (FSH). Studies have shown a significant increase in the frequency and the amplitude of LH release reflecting an increase in GnRH secretion with average/reduced FSH secretion, suggesting the presence of hypothalamic defects in PCOS [18, 19]. The elevated LH/FSH ratio is commonly observed in ovulatory women with polycystic ovary morphology (PCOM) [20]. The excessive hypothalamic GnRH secretion in PCOS patients shows a reduced sensitivity to inhibition by estradiol and progesterone [17]. Studies have also shown the role of neuropeptide kisspeptin coded by Kiss 1 gene as GnRH pulse generator. The GnRH neurons get a direct signal by Kisspeptin, which acts upstream of GnRH, to control pulsatile GnRH release [21]. Experimental studies in prenatally androgenized monkeys show the neuroendocrine dysregulation of the hypothalamic-pituitary-ovarian axis, resulting in increasing production of luteinizing hormone followed by increased ovarian androgen production [22]. Although androgen excess is a primary abnormality in PCOS, independent from hypothalamic–pituitary neuroendocrine dysregulation, studies so far have reported the dysregulation in the feedback loops between the hypothalamus-pituitary and the ovary.

## Steroidogenesis and hyperandrogenism

**Fig. 2:**
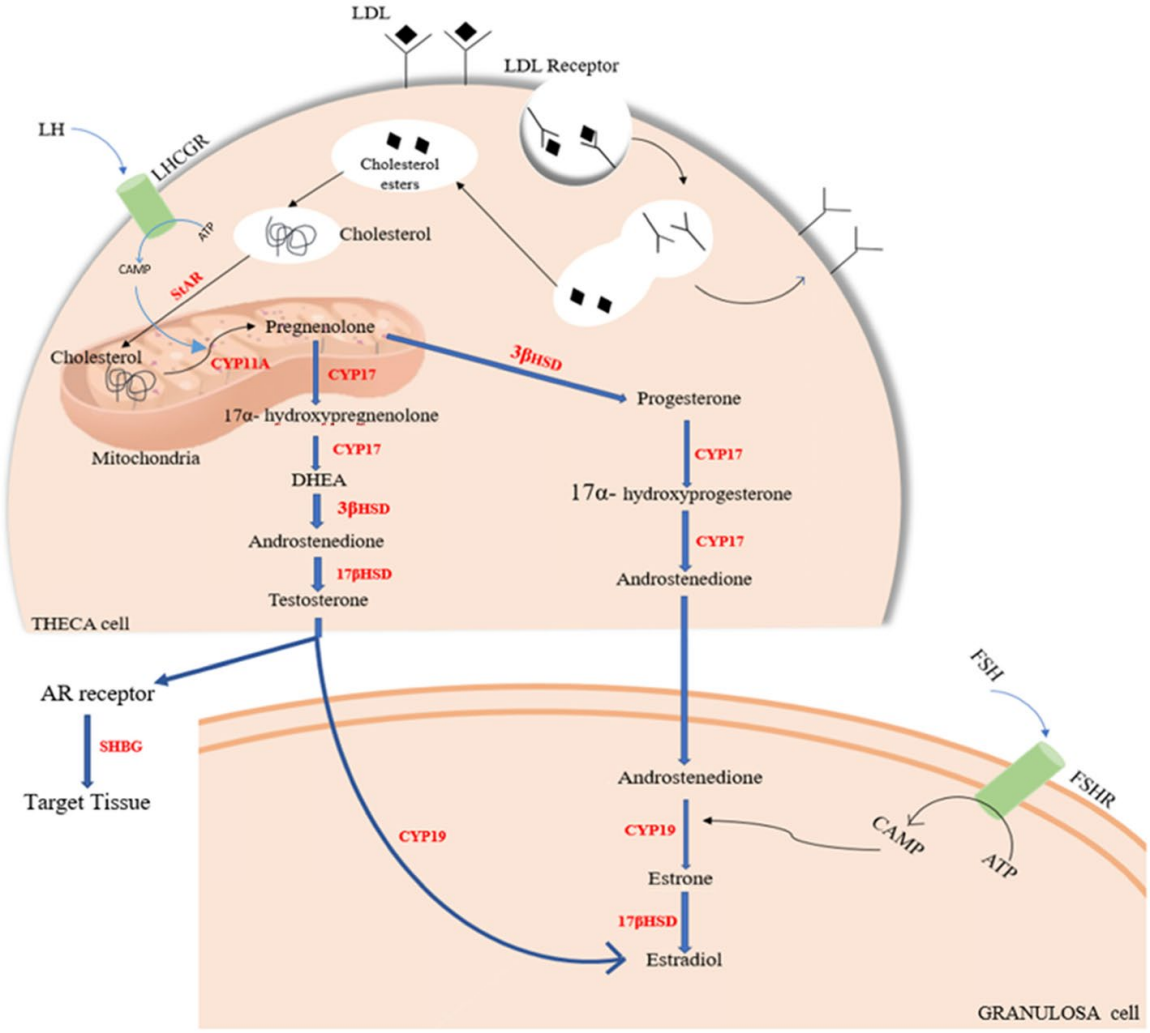
Schematic diagram of Steroidogenesis pathway and the enzymes involved in the biosynthesis. Abbreviations: LHCGR, luteinizing hormone/choriogonadotropin receptor; LDL, low-density lipoprotein; LDL receptor, low-density lipoprotein receptor; 3βHSD, 3β-hydroxysteroid dehydrogenase; StAR, steroidogenic acute regulatory protein; cAMP, cyclic adenosine monophosphate; ATP, adenosine triphosphate; FSH, follicle-stimulating hormone; FSHR, follicle-stimulating hormone receptor; LH, luteinizing hormone; CYP, Cytochrome P450; 17βHSD, 17β-hydroxysteroid dehydrogenase; DHEA, dehydroepiandrosterone; AR, androgen receptor; SHBG, sex hormone-binding globulin

The ovary is the major site for steroidogenesis, where the differentiation of theca cells and granulosa cells plays a vital role in follicular development and maturation. In a normally ovulating woman, the theca interna of the ovarian follicle and the adrenal cortex’s zona fasciculata significantly contribute to the secretion of androstenedione, and granulosa cells influence the conversion of androstenedione to estradiol under the activity of aromatase. Furthermore, the enzymes involved in the formation of androstenedione and estradiol are regulated by LH, FSH, and adrenocorticotrophic hormone (ACTH) in the ovary and adrenal glands [23–25]. The conversion of precursor cholesterol to biologically active steroid hormones is known as steroidogenesis. Steroidogenic enzymes, which include several cytochrome P450 enzymes (CYP), hydroxysteroid dehydrogenases (HSDs), and steroid reductases, carry out the biosynthesis of various steroid hormones, like androgens and estrogens [26]. The pre-requisite step that forms the precursors for other steroid hormones is the conversion of cholesterol to pregnenolone by CYP11A (cholesterol side-chain cleavage) and pregnenolone to progesterone by 3-hydroxysteroid dehydrogenase (3-HSD) specified in Fig. 2: Schematic diagram of Steroidogenesis pathway and the enzymes involved in the biosynthesis [27]. Under the influence of high pulse LH release, theca cells increase steroidogenic activity and upregulate the StAR, P450scc, 3-HSD, and CYP17, which produces androstenedione, which is further enhanced by increased levels of insulin commonly observed in PCOS women [28]. Insulin resistance and hyperinsulinemia lower the levels of sex hormone-binding globulin (SHBG), leading to an increase in androgen production [29]. Under the influence of pituitary FSH, androstenedione is converted to estrogen by aromatase present in granulosa cells [28]. In PCOS women, hyperactive ovarian theca steroidogenesis causes the overproduction of androgenic steroids, mainly 7-hydroxyprogesterone and androstenedione resulting in hyperandrogenism [30]. Furthermore, PCOS women have reduced aromatase activity and follicular development is impaired and arrested due to the relative decrease in FSH secretion, resulting in excess androgen accumulation and hyperandrogenemia [30]. Therefore, hyperandrogenism seems to play a crucial role in the pathogenesis of PCOS, contributing to the reproductive and metabolic aspects of the syndrome.

## Candidate genes involved in pathophysiology of PCOS

The increasing evidence of PCOS, hyperandrogenism, and metabolic alterations, and their hereditability is more observed in affected siblings of the family cluster than the general population [31–33]. There are several well-demonstrated biochemical abnormalities, despite the heterogeneity of PCOS, that can provide a reliable basis for adapting a candidate gene approach to the identification of susceptibility loci. So far, several genetic studies have identified almost 100 susceptibility genes related to PCOS. The relationship between target genes and disease risk variants is determined using linkage and association studies within the population or families. A transmission disequilibrium test (TDT) in affected siblings with hyperandrogenemia and PCOS-related traits predicted a strong association of follistatin, a nominal association of CYP11A1 gene, and a strong genetic association D19S884 allelic marker around INSR gene with PCOS [34]. Polymorphisms in genes involved in metabolic or regulatory pathways of steroid hormone synthesis, gonadotropin action, and insulin-signaling pathways have been investigated as PCOS susceptibility genes; however, the precise role of these susceptibility genes has not yet been to be determined [20, 35–38]. In contrast to candidate gene approaches that study relatively small samples, genome-wide association studies (GWAS) provide researchers with a more systematic, unbiased approach to exploring thousands of variants across the entire genome in both case and control individuals to discover the association of genetic variants in a complex disease like PCOS. Hence, 11 susceptibility loci mapping to DENND1A, THADA, LHCGR, FSHR, INSR, TOX3, YAP1, RAB5B, c9orf3, HMGA2, and SUMO1P1/ZNF217 have been identified in Han Chinese populations, which are involved in various pathways [38, 39]. Polymorphism in CYP11A, CYP17, CYP19, CYP21, βHSD, playing a role in the steroidogenesis pathway, results in the phenotypic expression of PCOS. Besides, the androgen receptor (AR) gene mediates the androgen level, and SHBG regulates the free serum androgen level; thus, all these genes may involve in the etiopathogenesis of PCOS. The candidate genes studies are the widely used technique to find the variants of the gene of interest and examined for correlation of androgen biosynthesis pathway and those affecting the secretion or action of insulin with PCOS etiology [40]. In addition, the name of the genes, the physiologic function affected by the genes, the studied population, and the type of single nucleotide polymorphisms or polymorphisms related with PCOS are detailed in this Table 1. Hence, in the below section, these genes, and their association with PCOS risk are described.

**Table 1:** Candidate gene polymorphism associated with the pathogenesis of Polycystic ovary syndrome

**Gene**	**Polymorphism**	**Studied population**	**Physiological function**	**Reference**
CYP11A	5’UTR (TTTTA)n pentanucleotide repeat	Samaritan	Hirsutism	[35]
	(TTTTA)_n_ Microsatellite	Caucasian	Hyperandrogenism	[41]
		Greek		[42]
	(TTTTA)_n_ Pentanucleotide repeat	Different	Hyperandrogenism	[43]
CYP17	-34 T/C polymorphism	British	Hyperandrogenism Insulin resistance	[44]
		American		[44]
		Korean		[44]
		Chinese		[45]
		Thai		[44]
		Indian		[46]
		Turkish		[47]
CYP19	rs2414096 polymorphism	African	Hyperandrogenism	[48]
		American		[48]
		Caucasian	Decreased aromatase activity	[48]
		Chinese		[49]
		Iranian	Increased E2 to T ratio	[50]
		Indian		[51]
		Iraqi		[52]
		Egyptian		[53]
		Japanese		[48]
		Chinese		[54]
	(TTTA)n Tetranucleotide repeat polymorphism	Greek	Hyperandrogenism	[55]
		Han Chinese		[56]
17βHSD	−71 A/G polymorphism	Greece	Hyperandrogenism	[57]
		Caucasian		[58]
		African-American		[59]
		Spanish		[60]
SHBG	TAAAA repeat Polymorphism	French	Hirsutism	[61, 62]
		Greek	Late menarche	[63]
		Croatian	Decrease SHBG level	[64]
		Slovenian		[65]
	rs1799941 & rs 727,428	Chinese	Insulin resistance	[66]
		American		[67]
	D327N	Mediterranean	Hirsutism	[68]
		French	Androgen excess	[62]
	E326K	Turkey	Metabolism of SHBG	[69]
AR	Short CAG repeat	Chinese	Hyperandrogenism	[70]
		Caucasian	Increased androgen sensitivity	[71]
				[72, 73]
	GGN polymorphism	Chinese	Hyperandrogenism	[74]
INSR	C/T polymorphism	Caucasian	Insulin resistance	[75]
		Chinese		[76]
		Korean		[77]
LHCGR	rs13405728	Han Chinese	Hyperandrogenism	[78]
	S312N	Sardinian		[79]
FSHR	rs6165	Turkish		[47]
	rs 6166	Italian		[80]
		Korean		[81]
		Chinese	Difference in FSH & PRL levels	[82, 83]
	rs2268361	Chinese	Folliculogenesis	[39]
GnRHR	rs104893836	Israeli		[84]
IL-1	rs1800587	Turkish	Biochemical, hormonal changes	[85]
		Caucasian		[86]
		Caucasian		[86]
	rs16944	Chinese		[87]
PPARG	Pro12Ala	Korean	Abdominal obesity	[88]
			Metabolic dysfunction	
			BMI	
		European	BMI	[89]
		Asian		[89]
		Caucasian	Insulin resistance Hirsutism	[90–94]
VDR	rs1544410, rs7975232	South Indian	Biochemical, metabolic, endocrine parameters	[95]
		Iranian		[96, 97]
	rs10735810, rs731236	Turkish		[98]
	rs757343	Austrian		[99]
FTO	rs9939609	Chinese	Obesity	[100, 101]
		UK		[102]
		Finland		[102]
		South Brazilian		[103]
		Caucasian		[104]
		East Asian		[104]
ACE	ACE I/D polymorphism	Caucasian	Hyperandrogenism	[105]
		Chinese		[106]
		Turkey		[107, 108]

In addition, the name of the genes, the physiologic function affected by the genes, the studied population, and the type of single nucleotide polymorphisms or polymorphisms related with PCOS are detailed in this Table 1

*Abbreviations*: *17βHSD* 17β-Hydroxysteroid Dehydrogenase, *SHBG* Sex hormone binding globulin, *AR* Androgen receptor, *INSR* Insulin receptor, *FSHR* Follicle stimulating hormone receptor, *GnRHR* Gonadotropin releasing hormone receptor, *LHCGR* Luteinizing hormone/chorionic gonadotropin receptor, *IL-1* Interleukin-1, *PPARG* Peroxisome Proliferator Activated Receptor Gamma, *VDR* Vitamin D receptor, *FTO* Fat Mass and Obesity-Associated Protein, *ACE* Angiotensin Converting Enzyme

## CYP11A

CYP11A is the side-chain cleavage enzyme (P450scc) located on chromosome 15q23-q24 [109] catalyses the conversion of cholesterol to pregnenolone, which is the first and rate-limiting enzymatic step in the biosynthesis of all steroid hormones [110]. P450scc is expressed in the ovary, more specifically in the theca interna and the granulosa cells of ovulatory follicles [111]. Apart from the ovary CYP11A is also expressed in the adrenal cortex, testis, and placenta [112]. There is an unusual exon/intron junctional sequence starting with GC in the sixth intron in the CYP11A gene which is at least 20 kb with nine exons split by eight introns [113]. According to the linkage review, a CYP11A 5′ UTR (TTTTA)_n_ pentanucleotide repeat polymorphism has a robust allelic association with hirsute PCOS patients [35]. Studies carried in the different ethnic groups showed varied association of these pentanucleotide repeat alleles with PCOS susceptibility. In the Caucasian population, a recent meta-analysis found a clear connection between the microsatellite (TTTTA)_n_ repeat polymorphism of CYP11A and an increased risk of PCOS [41]. The allelic variants of CYP11A and its polymorphism associated with serum testosterone level might be associated with androgen excess and hyperandrogenemia [42]. In the United States, South India, and Greece, a repeat polymorphism (TTTTA)_n_ in the promoter region of the CYP11A gene has been linked to PCOS in contrast to cases reported in Spanish, Chinese, Argentinian, Indian showed no association in women with PCOS [114–116]. The meta-analysis findings revealed a connection between PCOS and a pentanucleotide repeat polymorphism in the CYP11A1 promoter [43]. Furthermore, the association of this gene with hirsutism and no significant association with ovulatory function indicates that CYP11a predominantly has a role in the development of hirsutism in PCOS [35]. Therefore, knowing the crucial role of this gene in ovarian steroidogenesis, all the studies imply the CYP11A gene as a possible genetic biomarker playing a major role in the pathogenesis of PCOS.

## CYP17

CYP17 (P450c17), located on chromosome 10q24.3 [117], catalyzes two mixed-function oxidase reactions utilizing cytochrome P450 oxidoreductase and the microsomal electron transfer system [118]. The 17-hydroxylyase and 17-lyase activity of the P450c17 enzyme catalyses the conversion of pregnenolone to 17-hydroxypregnenolone and progesterone to 17-hydroxyprogesterone, followed by the cleavage of the 17–20 bond to create the C19 steroids dehydroepiandrosterone and androstenedione [119]. The expression of CYP17 is observed in all steroidogenic tissues; however, in the adrenal gland and placenta, some species-related differences in the expression of the enzyme are reported. In the ovary, the expression of CYP17 is limited to theca cells that are the site of androgen production [120, 121]. Although granulosa cells and luteal cells do not express CYP17, a recent report suggests that human luteinized granulosa cells in culture do express CYP17 [122]. The P450c17 enzyme has been shown to have increased activity and expression in the ovarian theca cells of PCOS women, along with the increased transactivation of the CYP17 promoter [123, 124]. Studies have also shown the dysregulation of CYP17 expression of mRNA stability in PCOS theca cells [125]. Numerous mutations have been reported in the CYP17 gene and many studies have explained the polymorphism in this gene [126–129]. Studies have reported that the polymorphism in the 5’UTR region which involves a single base-pair change (T-C), at a − 34 position in the promoter region, regulates the expression of CYP17 and androgen levels, creating an additional Sp1 transcription factor binding site [44, 128]. However, in previous studies conducted in British, American, Korean, Chinese, Thai, and Indian women with PCOS and Turkish adolescents, this polymorphism was not found to be a significant risk factor for PCOS growth [47]. Even though the CYP17 gene does not appear to be a candidate gene for PCOS pathophysiology, it plays a predominant role in developing hyperandrogenic phenotype and insulin resistance in women with PCOS [45, 46, 130]. Therefore, more detailed research is needed to understand the exact mechanism and role of the gene in the etiology of PCOS.

## CYP19

CYP19 (P450arom), located on chromosome 15q21.1 [131, 132], catalyzes the transformation of the C19 androgens, androstenedione, and testosterone, to the C18 estrogens, estrone, and estradiol [133, 134]. The primary sites for the expression of P450arom are in the ovary, adipose stromal cells, placenta, bone, and various fetal tissues [135, 136]. In the ovary, the granulosa cells of preovulatory follicles show higher expression of P450arom than do small follicles as well as in the corpus luteum of ovulatory women [137, 138]. Many studies have reported the deficiency of aromatase activity in patients with hyperandrogenism [139, 140]. Furthermore, there is a significant decrease in the activity of P450arom (irrespective of the BMI in women with PCOS) in both lean and obese women with PCOS [141]. Studies have reported that the reduced expression of CYP19A1 by the hypermethylation of the promoter region decreases the aromatase enzyme’s overall activity in women with PCOS varies [142]. The SNP of CYP19 rs2414096 showed significant association with reduced aromatase activity, increased estradiol to testosterone ratio (E2/T), hyperandrogenic phenotype, and PCOS development in African, American, Caucasian [48], Chinese [49], Iranian [50], Indian [51], Iraqi [52], Egyptian [53]. However, the association of CYP19 rs2414096 was not found statistically significant in Japanese women with PCOS [48]. Moreover, a tetranucleotide repeat polymorphism (TTTA)*n* in the CYP19 gene with short alleles inhibits aromatase activity, resulting in hyperandrogenism and its association with increased testosterone levels, high LH: FSH ratios in women with PCOS has been reported [54–56, 143]. Studies have been reported increased levels of testosterone from follicular fluid of PCOS women, significantly reduce the expression of the aromatase enzyme in luteinized granulosa cells [144]. Therefore, different studies showed a significant association of aromatase enzyme in hyperandrogenism, and androgen biosynthesis represents a pivotal role of CYP19 as a susceptible gene in PCOS development.

## CYP21

CYP21 (P450c21) located on chromosome 6p21.3 [145]. The 21-hydroxylase enzyme catalyzes the hydroxylation of C21 steroids converting progesterone and 17-hydroxyprogesterone into 11-deoxycorticosterone and 11-deoxycortisol [146]. The major site for the expression of CYP21 is only in the adrenal cortex that is vital for the synthesis of adrenal-specific steroids, the glucocorticoids, cortisol, and corticosterone, and the mineralocorticoid, aldosterone [147, 148]. The expression of the P450c21 enzyme is not detected in the kidney, liver, testis, or ovary [149]. Studies have reported an increased frequency of heterozygosity for CYP21 gene mutation in women with symptomatic hyperandrogenism, premature pubarche, and PCOS-like phenotype [150–152]. Furthermore, the frequency of heterozygosity for CYP21 mutations was found to be significantly higher in Spanish women with hirsutism, in both American and Greek children with premature pubarche, and in American adolescent girls with hyperandrogenism [150, 153, 154]. Overall, the CYP21 gene and its mutations do not appear to play a significant role in the predisposition of PCOS; however, it can play a minor role that further studies will solve.

## 3βHSD

The 3β-Hydroxysteroid Dehydrogenase (3βHSD) enzyme is located on chromosome 1p13.1 [155]. The 3βHSD enzyme is essential for the biosynthesis of active steroid hormones, catalyzing the dehydrogenation and isomerization reaction converting delta5–3-β-hydroxysteroids, pregnenolone, and dehydroepiandrosterone into delta4–3-ketosteroids, progesterone, and androstenedione [156]. The expression of the 3βHSD isoform is tissue specific. The isoform 3βHSD II is expressed in the adrenal gland, ovary, and testes [157]. Studies have reported that the deficiency of the 3βHSD enzyme is associated with mild virilization and irregular or absent ovulation [158]. In Addition, the enzyme’s deficiency in hyperandrogenic females is linked to insulin resistance and LH hypersecretion in PCOS patients [159, 160]. There is a specific decrease in the expression of the 3βHSD gene in luteinizing granulosa cells with large follicle size in women with polycystic ovaries [161]. The deficiency of the 3βHSD gene and its association does not seem to play a significant role in PCOS development. Therefore, more research needs to be incorporated.

## 17βHSD

The 17β-Hydroxysteroid Dehydrogenase (17βHSD) enzyme on chromosome 10p14-p15 [162] plays an essential role in steroidogenesis. The 17βHSD enzymes catalyze the final step in the biosynthesis of active gonadal steroid, the conversion of androstenedione to estradiol and testosterone [159]. Type 5 of the 17βHSD gene is exclusively expressed in the ovary and adrenal gland [58]. Immunohistochemical studies have shown the expression of the 17βHSD type 5 gene in ovarian theca cells and corpus luteum [163]. Studies reported the increased frequency of −71A/G polymorphism in the 17βHSD type 5 promoter region and its association in Caucasian women with PCOS. Furthermore, it is observed that this SNP increases the 17βHSD type 5 promoter activity and its affinity for the transcription factors Sp1/Sp3. Some menstrual irregularities are also observed due to the accumulation of androstenedione due to the deficiency of this enzyme [164]. The −71A/G polymorphism in the 17βHSD type 5 gene is also associated with hyperandrogenemia and increased serum testosterone levels in women with PCOS but does not contribute to the pathophysiology of PCOS [59]. However, subsequent studies failed to identify the association between this SNP and hyperandrogenemia phenotype in African American, Caucasian, and premature pubarche in Spanish [57, 59, 60, 164]. Another polymorphism of 17βHSD type 5 gene, rs1937845 and rs12529, shows increased serum testosterone level, homeostasis model assessment of *β*-cell function (HOMA-B) index indicating the association of insulin resistance with hyperandrogenism in Chinese women [165]. 17βHSD type 5 polymorphism does not influence the effect of oral contraceptive pills in Brazilian women with hirsutism and androgen excess [166]. Therefore, the polymorphism of the 17βHSD type 5 gene may play a crucial role in the development of hyperandrogenemia and insulin resistance and can be regarded as a candidate gene for the etiopathogenesis of PCOS.

## SHBG

The Sex hormone-binding globulin (SHBG) gene is located on chromosome 17p13.1 [167]. SHBG is primarily produced by hepatocytes, binds to androgens and estrogen with high affinity, thus, controls the levels of sex hormones within the circulation and regulates the access of target tissues to androgens [168, 169]. The prime expression of SHBG receptors (RSHBG) is observed in sex-steroid-dependent cells and tissues which include ovaries, endometrium, colon, prostate, hypothalamus, breast, placenta, liver, epididymis, immune cells, and cardiomyocytes [170]. PCOS women are attributed with increased androgen levels and often present with insulin resistance and compensatory hyperinsulinemia, which inhibits the hepatic synthesis and secretion of SHBG resulting in low circulating SHBG concentrations [171]. Studies have reported that low serum SHBG levels in PCOS women result in hyperandrogenic symptoms such as hirsutism, acne, androgenic alopecia, and virilization [172–175]. Furthermore, some common genetic variations in the SHBG gene also influence circulating SHBG levels and may contribute to the PCOS phenotype [176, 177]. Studies have reported two novel coding region mutations, including one with abnormal glycosylation and the other is truncated SHBG synthesis in women, resulting in low SHBG levels and increased circulating free testosterone concentrations [178]. The correlation between a longer TAAAA repeats polymorphism and later menarche and lower SHBG levels in hirsute French women suggests that SHBG polymorphisms may play a genetic role [61, 62]. In Greek PCOS women, longer allele genotype showed a positive association with lower SHBG levels [63]. The long TAAAA repeat alleles, on the other hand, did not display any association with PCOS aetiology in Croatian, Slovenian, or Chinese women [64–66]. Long SHBG alleles in combination with short CYP19 alleles resulted in low SHBG levels and increased testosterone levels, and elevated FAI, DHEAS, and T/E2 ratios in Greek women with PCOS [179]. Overall, the meta-analysis findings indicated insufficient evidence to draw a definitive connection between the TAAAA repeat polymorphism and PCOS risk, implying that it might not be a good predictor of PCOS risk [180]. Four single nucleotide polymorphisms (SNPs; rs1799941, rs6257, rs6259, and rs727428) have been identified as predictors of the development of type 2 diabetes in men and women and as modifiers of serum SHBG concentrations [181, 182]. However, a family-based and case-control study conducted in American and Mediterranean women with PCOS showed no direct association with PCOS risk. SNPs rs1799941 and rs727428 in the SHBG gene influenced serum SHBG concentrations after controlling for BMI and indexes of androgen excess and insulin resistance [67, 68]. Several studies have been published. Exon 8 contains a functional missense polymorphism that induces an amino acid transition from aspartic acid to asparagine (D327N), which causes a delay in SHBG half-life and affects SHBG metabolism [62]. On the other hand, E326K, another missense polymorphism on exon eight, lowers SHBG levels and influences the SHBG metabolism independent of BMI, androgen, and insulin-related traits in PCOS women [69]. A recent study conducted in Bahraini women reported that specific SHBG variants affecting the SHBG concentrations and SHBG haplotypes spanning six polymorphisms were linked to increased or decreased PCOS susceptibility [183]. Thus, SHBG can be considered as a candidate gene playing a central role in the pathophysiology of PCOS.

## AR

Androgen effects are facilitated by androgen receptors (AR). The AR gene is located on the X-chromosome at Xq11–12 and with a genetic polymorphism in exon one characterized by a CAG trinucleotide repeat encoding polyglutamine restudies [184]. Increased androgen levels show association with inhibition of follicle development, anovulation, menstrual irregularities, and appearance of micro cysts in the ovaries [185, 186]. Exposure to intrauterine androgens in experimental models leads to the development of PCOS phenotype in adult life [187]. Theca interna cells of preantral follicles, granulosa cells of preantral and antral follicles, and both theca and granulosa cells of dominant follicles have all been found to contain AR [188]. The genetic polymorphism in the AR gene in exon one with CAG repeats indicates the association between AR activity and PCOS prevalence [189]. Studies have reported an increased frequency of short AR CAG repeats in PCOS women and may contribute to PCOS onset in both Chinese and Caucasian populations [70, 71]. Furthermore, in PCOS patients, this polymorphism causes AR upregulation and increased androgen sensitivity [72, 73]. However, no association of AR CAG repeat lengths in Indian [190] Slovene [191], Korean [192], and Croatian [193], was reported in PCOS women. Furthermore, few studies have also reported an association of CAG repeats length with higher serum testosterone levels in PCOS women [192–195]. Another study carried by Hickey et al. showed preferential expression of longer CAG repeats in infertile Australian PCOS women compared to fertile PCOS women that were also found positively correlated with serum testosterone levels. Additionally, a study comparing PCOS families found that sister pairs with diverse patterns of XCI were more likely to display clinically varied PCOS symptoms than sister pairs with identical XCI profiles, emphasizing the relevance of XCI in the pathogenesis of PCOS [196]. In contrast, Mifsud et al. found lower testosterone levels in PCOS patients with short CAG repeats [73]. On the other hand, a study conducted by Westberg et al. in premenopausal Swedish women found higher levels of serum androgens with fewer CAG repeats than women with longer repeats [197]. In addition to influencing AR expression, the XCI pattern can influence the expression of BMP15 (Xp11.2), a gene implicated in preovulatory follicular development [198]. Surprisingly, BMP15 increases FSHβ subunit transcription and secretion while not affect LH expression [199]. Calvo et al. investigated the relationships between AR-CAG allele length, XCI pattern, and hirsutism and compared AR CAG-BM levels to hormone (such as DHEAS) levels but found no significant difference links [200]. As a result, we believe the XCI pattern alters LH and FSH levels by directly altering the expression of gonadotropins or other genes required for folliculogenesis. Studies have also reported a significant GGN polymorphism and rs6152G/A polymorphism with Chinese PCOS women [74, 201]. The meta-analysis showed no significant association between CAG repeat lengths at AR and PCOS risk, unlikely to be the primary determining factor in PCOS etiology [202, 203]. Thus, androgen excess has a vital role in the developing hyperandrogenic phenotype in PCOS women and the pathophysiology of PCOS.

## StAR

The Steroidogenic acute regulatory protein (StAR) is located on the short arm of chromosome 8p11.2 [204]. StAR protein acts as a transporter protein, which plays a major role in the transportation of cholesterol from the outer to the inner mitochondrial membrane in the first step of the steroidogenesis pathway [205, 206]. The gene expression of steroidogenic enzymes including StAR was studied from granulosa and theca cells of women with PCOS. The follicles of theca cells showed increased expression of StAR in comparison to the size-matched control follicles which indicates the hyperstimulation of theca cells producing excessive amounts of androgens. However, in granulosa cells, there were no changes in the expression of StAR in follicles of PCOS women from control follicles, indicating the increased LH responsiveness of granulosa cells in PCOS women, which may contribute to arrested follicle development [207]. Another study conducted by Kahsar-Miller et al. showed no changes in expression of StAR in PCOS ovaries compared to normal healthy ovaries [208]. In Iranian PCOS women, however, no correlation was found between seven StAR SNPs [209].

## INSR

The insulin receptor (INSR) gene is located on the short of chromosome 19 [210], which plays a significant role in insulin metabolism. The HAIR-AN syndrome (hyperandrogenism, insulin resistance, and acanthosis nigricans), a subset of PCOS marked by extreme insulin resistance, demonstrates the significance of insulin signaling in PCOS [211]. Insulin resistance may stimulate LH hypersecretion in the pituitary, increased testosterone production in theca cells, and P450scc activity in granulosa, and disturbs the follicular maturation, resulting in PCOS [212]. The polymorphism, C/T SNP at His1058 in exon 17 of the INSR gene has been significantly associated with Caucasian and Chinese PCOS women [75, 76]. However, in the Korean population, this polymorphism failed to confirm the association [77]. On the other hand, a novel T/C polymorphism at Cys1008 in exon 17 was associated with decreased insulin sensitivity in Chinese PCOS women [213]. Besides, linkage analysis studies predicted a microsatellite marker D19S884, located on chromosome 19p13.2, close to INSR gene associated with PCOS and was considered as a candidate gene [34, 214]. Other SNPs includes rs225673 in intron 11 and rs8107575, rs2245648, rs2245649, rs2963, rs2245655, and rs2962 around exon 9 in the INSR gene have shown an association with PCOS. However, the impact on gene expression or its association with underlying genetic variation is still uncovered [215, 216]. The results of meta-analyses showed no significant association between SNPs rs1799817 or rs2059806 with the development of PCOS. Nonetheless, SNP rs2059807 can be considered as a candidate risk factor for PCOS development [217]. Hence, all these studies so far suggest the association of the genetic variant in exon 17 of INSR with the pathophysiology of PCOS and INSR gene, being a crucial component of the insulin signaling pathway, could be a plausible candidate gene for PCOS.

## LHCGR

The luteinizing hormone/choriogonadotropin receptor (LHCGR) gene, mapped on chromosome 2p16.3 [218] is a G-protein coupled receptor expressed predominantly in the granulosa cells of preovulatory follicles and is responsible for ovulation in response to the mid-cycle LH surge [219]. Inactivating mutations of LHCGR cause increased LH levels, menstrual irregularities, and infertility in women, while activating mutations cause hyperandrogenism [220]. A recent GWAS study identified the 2p16.3 region containing LHCGR loci to be associated with PCOS in Han Chinese and European populations [78, 221]. The LHCGR rs13405728 variant showed association with PCOS in Han Chinese women. However, it failed to explain association in European-derived and Caucasian population [78, 222–224], indicating that racial/ethnic background contributes to PCOS development. S312N, a nearby SNP in exon 10 (rs2293275) of LHCGR gene induces an amino acid substitution in the Sardinian population, was linked to PCOS [79]. The data obtained from the genomic study of LHCGR describes racial/ethnic background. Hence, independent ethnic research is needed to rule out the connection between gonadotropin receptor variants and an increased risk of PCOS.

## FSHR

The Follicle-stimulating hormone receptor (FSHR), located on chromosome 2p21, is a G protein-coupled receptor, expressed in granulosa cells similarly to LHCGR [225]. FSHR stimulates oogenesis, follicle development, and gametogenesis, resulting in follicular maturation and proliferation of granulosa cells on binding with FSH [226]. Inactivating mutation in the FSHR gene results in hypogonadotropic hypogonadism and induces arrest of follicle development at the preantral stage [227]. A recent GWAS study reported the association of the FSHR gene with PCOS in the Han Chinese population and European-derived population [39, 78, 221]. The two variants in exon 10 of the FSHR gene rs6165 (Thr307Ala) and rs6166 (Asn680Ser) have been studied in association with PCOS [47, 80–83]. However, the meta-analysis results showed the association of SNP rs6166 (Asn680Ser) showed with PCOS women, while, the SNP rs6165 (Thr307Ala) failed to show any association with PCOS [228]. Another polymorphism, rs2268361 showed an association with PCOS in the Chinese population [39] but not in Dutch [229]. The relationship between the genotype of the FSHR variants and PCOS and how exactly it contributes to PCOS development is not clear. Hence, the genetic variants of the FSHR gene studied irrespective of the race difference can be considered as a risk factor for PCOS.

## GnRHR

Gonadotropin-releasing hormone receptor (GnRHR) is a G-protein coupled receptor found in the anterior pituitary’s gonadotroph membrane and many extra-pituitary tissues such as the ovary, placenta, breast, and cancer tissues [230, 231]. GnRH on binding with its receptor GnRHR activates the phosphatidylinositol-Ca2+ second messenger system and modifies LH and FSH synthesis and secretion [232]. The polymorphism of the GnRHR gene was detected using PCR-RFLP assay and the findings revealed that TCC, CCC, and CCT haplotypes increased the risk of PCOS, while TTT, TCT, and TTC haplotypes reduced the risk [233]. Three sisters from a consanguineous family with PCOS were examined in a recent genome-wide study using whole-exome sequencing. Sanger sequencing of the rs104893836 variant in the first exon of the GnRHR and suggested that genetic variation in the hypothalamus-pituitary axis seems to play role in the pathogenesis of PCOS [84]. The genetic alteration in GnRH and its receptor might play a role in the development of PCOS. However, susceptible variants in this gene as a PCOS risk factor are still not discovered.

## IL-1

Interleukin 1 (IL-1) is a crucial multifunctional proinflammatory cytokine composed of three distinct cytokines: IL-1α, IL-1β, and the physiologic antagonist IL-1 receptor antagonist (IL-1RA) [234]. IL1α and IL1β are located on chromosome 2q14.2 within a 430 kb area [235]. In reproductive biology, IL-1 is thought to alter ovulation, fertilisation, and implantation due to its inflammatory traits [236]. According to research, the polymorphism rs1800587 (−889C/T) reduces IL-1 gene transcription through altering IL-1α protein expression in ovarian tissue in Caucasian population [85, 86]. Moreover, the first association study of the two IL-1β polymorphism rs 16,944 (−511C/T) and rs 1,143,634 (+ 3953 C/T) and PCOS development was conducted by Kolbus et al. in Caucasian population but failed to find a correlation. However, another study conducted in Chinese population showed that the rs 16,944 (−511C/T) polymorphism showed association with developing PCOS by somehow altering the IL-1β production. However, no association was observed while studying the rs 1,143,634 (+ 3953 C/T) [86, 87]. Hence, these findings suggest that IL-1 family gene polymorphism may be an influential marker for the risk of PCOS.

## PPARG

Peroxisome proliferator activated receptor gamma (PPARG) is a ligand-activated transcription factor located on chromosome 3p24.2-p25 [237]. It impacts adipocyte differentiation, insulin sensitivity, lipid metabolism, and the development of atherosclerosis [238]. PPARG has many single nucleotide polymorphisms (SNPs), the most studied of which is PPARG Pro12Ala. Studies have showed the association of Pro12Ala polymorphism with abdominal obesity in Korean PCOS women with metabolic dysfunction since PPARG plays an essential role in adipose tissue metabolism [88]. The Ala allele carriers reported significant higher BMI waist circumference, waist to hip ratio and sum of skinfolds than non-carriers in PCOS cohort [239]. A meta-analysis conducted in European and Asian population reported a positive relationship between Pro12Ala polymorphism and BMI [89]. In addition, some studies found a significant increase in insulin sensitivity (lower HOMA-IR), as well as lower fasting insulin and glucose levels in Caucasian population [90–93] and a lower hirsutism score in PCOS women carrying the Pro12Ala G allele [94]; however, others, found no link between fasting glucose and insulin or changes in HOMA-IR in PCOS women carrying the Pro12Ala G allele [240–243]. Even though considerable research on Pro12Ala polymorphism in diverse ethnic populations of PCOS women have been undertaken, the majority of the results have been inconsistent, if not wholly contradicting.

## KISS 1

Kisspeptin (KISS) is a neuropeptide located on chromosome 1q32 [244]. Kiss 1 gene stimulates the activity of GPR54, a G protein–coupled transmembrane receptor present in GnRH neurons, and hence increases LH levels [245]. Kisspeptin has been implicated in the control of the HPG axis in numerous studies since its discovery, at the cell, animal, and even human levels [246–248]. In specific trials, kisspeptin treatment has been shown to result in an almost 2-fold increase in LH levels, with a minor or non-existent increase in FSH levels [249–251]. Additionally, Kisspeptin has been shown to have a direct influence on GnRH neurons upstream in terms of depolarization directly, increased firing rate, and up-regulated expression of GnRH mRNA, which explains the elevated LH/FSH ratio found in prior studies [252–255]. Although the primary research has revealed a possible link between the KISS1 system and the HPG axis, it is still unclear whether the plasma/serum kisspeptin concentration is higher in PCOS women than in general. Kisspeptin levels were more significant in PCOS women than in controls in some research [256–258], while other studies found similar or negatively linked results [259, 260]. As a result, plasma/serum kisspeptin levels are likely to be related with serum LH levels, and therefore with the pathophysiology of PCOS.

## VDR

The Vitamin D receptor (VDR) Gene is located on chromosome 12q13.11 [261]. The VDR belongs to the nuclear receptor superfamily and is found in a various tissues, including the intestine, kidney, parathyroid gland, pancreatic beta cells, and bones, all of which are important in calcium homeostasis maintenance. The active form of vitamin D, 1,25 (OH)2-D3, regulates gene transcription in target organs by binding to the nuclear vitamin D receptor (VDR). In addition, for optimal VDR-DNA interaction, the VDR forms a heterodimer with the retinoid-X receptor (RXR) [262]. It is also expressed in human ovarian tissue and endometrium, and it has been shown to play a role in the steroidogenesis of sex hormones [263–265]. Irregularities in calcium balance may disturb follicular growth in women, affecting the aetiology of PCOS [266]. Although studies demonstrate that vitamin D deficiency might promote metabolic syndrome and insulin resistance in PCOS patients, it is unclear if vitamin D is associated with endocrine and reproductive parameters in PCOS patients [264, 267]. Several studies on VDR gene polymorphisms revealed a link between VDR BsmI (rs1544410),), ApaI (rs7975232), FokI (rs10735810), and TaqI (rs731236) and PCOS risk in South Indian women and Iranian women [95, 96, 268]. Ranjzad et al. also looked at the relationship between the FokI, BsmI, ApaI, TaqI, and Tru9I (rs757343) polymorphisms and biochemical and metabolic parameters in Iranian PCOS women. The findings demonstrated substantial relationships between lower levels of sex hormone binding globulin (SHBG) and both VDR BsmI “GG” and adiponectin (ADIPOQ) BsmI “CC” genotypes, implying that the “G” allele is a risk factor for PCOS in homozygotes [97]. Bagheri and colleagues investigated the FokI and BsmI variants of the VDR gene in the genetic predisposition to PCOS in Iranian and Azeri Turkish women. Their findings revealed no statistically significant differences in PCOS susceptibility in the examined group [98]. Furthermore, Wehr and colleagues performed a cohort analysis in Austrian women with PCOS to assess the relationship between VDR polymorphisms and PCOS susceptibility. They found no link between VDR BsmI, FokI, and TaqI polymorphisms and anthropometric, endocrine, or metabolic parameters [99]. According to the findings of many studies, the association between VDR gene polymorphisms and PCOS in different ethnicities is debatable. However, it may play a significant role in the pathophysiology of PCOS.

## FTO

The human Fat Mass and Obesity-Associated Protein (FTO) gene is found on chromosome 16q12.2 and is expressed in nearly all tissues [269, 270]. The protein encoded by the FTO gene is a 2-oxoglutarate-dependent nucleic acid demethylase involved in energy metabolism [271]. A genome-wide association analysis published in 2007 found that FTO is linked to body mass index (BMI) and obesity [269]. Obesity is a prevalent feature in PCOS patients, with more than half of all PCOS cases being overweight or obese [272]. A common single nucleotide polymorphism (SNP) (rs9939609) in the first intron of the FTO gene with a T to A change has recently been extensively researched in PCOS women. However, the results of various studies are conflicting. Studies found a strong correlation between FTO and PCOS in the Chinese, UK, Finland, and South Brazilian populations [100–103], while others revealed a link between FTO and BMI in PCOS women, although they do not appear to have a significant role in the reproductive phenotypes of PCOS [273–275]. Cai et al. found that the FTO rs9939609 polymorphism was linked with PCOS risk among East Asians but not in the Caucasian population [104]. As a result, it is fair to speculate that the FTO gene may have a role in the pathogenesis of PCOS via BMI and/or obesity.

## RXR

Human sebocytes express retinoid X receptors (RXRs), members of the steroid/thyroid hormone superfamily [276]. Retinol is critical for female reproduction, and retinoids have been implicated in ovarian steroidogenesis, oocyte maturation, and corpus luteum development [277, 278]. Retinoids have been shown to increase steroid hormone production in peripheral steroidogenic tissues. Retinoid therapy elevated the expression of steroidogenic acute regulatory protein (StAR) in mice Leydig cells, resulting in steroidogenesis potentiation [279]. All-trans- and/or 9-cis-retinoic acid enhanced gene expression of StAR, CYP17A1, and P450scc, as well as testosterone and dehydroepiandrosterone synthesis in human ovarian thecal cells [280]. When PCOS cell extracts were compared to standars extracts, the conversion of retinol to retinaldehyde was enhanced, indicating that the enzymes responsible for retinol metabolism are present in theca cells and may be changed in PCOS [281]. RXR boosted the expression of CYP19, a crucial regulator in oestrogen synthesis, and increased the synthesis of estradiol, which protects hippocampus neurons against OGD and inflammatory stimuli, demonstrating that RXR is responsible for CYP19 expression [282]. These findings revealed that retinoids had a significant impact on theca cell androgen production as well as the expression of steroidogenic enzyme genes. More research is needed to understand the pattern of expression of enzymes involved in retinol metabolism/retinoid production in ovarian cells, as well as their functional significance in retinoid action in PCOS.

## VEGF

The Vascular Epithelial Growth Factor (VEGF) is a homodimer glycoprotein that is expressed in granulosa and thecal cells and is known to play a role in the pathophysiology of PCOS [283]. It is involved in angiogenesis, follicular vascularisation, and intra-follicular oxygenation, and hence influences follicle maturation, oocyte quality, fertilisation, and embryo development [11, 28, 284]. PCOS is associated with increased stromal vascularity, which may be due to a dysregulation of numerous angiogenic factors, including VEGF. Daghestani et al. reported that VEGF levels in obese PCOS women were four times greater than in non-PCOS obese women, consistent with prior research indicating higher levels of VEGF in PCOS patients [285, 286]. As a result, the evidence so far suggests that VEGF may have a role in the aetiology of PCOS.

## ACE

Angiotensin converting enzyme (ACE), a critical factor in the conversion of Angiotensin I to Angiotensin II, is found in a various organs, including the ovaries. ACE and its products, in addition to regulating blood pressure and fluid balance, play an essential role in regulating ovarian function through follicular development, oocyte maturation, ovulation, and follicular atresia [287]. The inter-individual variability in plasma ACE concentration has been linked to an insertion (I)/deletion (D) polymorphism involving a 287-bp DNA sequence located in intron 16 of the ACE gene, known as the ACE I/D polymorphism [288]. A recent meta-analysis found a positive association between this polymorphism and PCOS risk in Caucasians, but no such association in Asians [289]. Koika et al. discovered a positive link between I/D polymorphism and PCOS in cases of hyperandrogenism but not in situations of non-hyperandrogenism [105]. Another study in a Chinese population discovered that the DD genotype was related to higher testosterone concentrations when compared to the II genotype [106]. Moreover, similar associations were discovered for fasting insulin and homeostatic model assessment for insulin resistance (HOMA-IR) in PCOS patients in Turkey [107, 108]. Furthermore, obese women with PCOS have greater total renin levels than age- and BMI-matched controls, but not ACE activity or aldosterone levels [290]. Based on considerable research conducted in various ethnic populations, the presence of a relationship between ACE I/D polymorphism and PCOS is debatable. This shows that, whereas I/D polymorphisms in the ACE gene were not the major etiological cause, they may be linked to worsened clinical symptoms of PCOS.

## Conclusion and future perspective

Polycystic ovary syndrome remains a complex endocrine paradox characterized mainly by surplus androgen production resulting in metabolic and gynecological concerns in affected individuals. The fact that 70% of women diagnosed with PCOS go on to become infertile makes it a concerning issue. With infertility on the rise and PCOS as a significant cause in women, early detection and treatment play critical roles in improving quality of life. As a result, we tried to look for unique polymorphisms of the chosen candidate gene that may be employed in the diagnosis and screening of PCOS. Although androgen excess is the primary cause of PCOS pathogenesis, the brain dysfunction route, which encompasses the hypothalamus-pituitary-ovarian axis, could also be the cause of PCOS. It is difficult to determine due to inhibition in the feedback loops involving the hypothalamus, pituitary, and ovary and should be investigated further to determine the aetiopathogenesis of PCOS. Hyperandrogenism can be studied by inducing PCOS phenotypes in fetal, neonatal, and prepubertal giving excess androgenic treatments to animal models. Another mechanism is using transgenic models for studying neuroendocrine dysfunction (HPO axis). Furthermore, obesity plays a vital part in the aetiology of PCOS, and most individuals with the condition are overweight or obese; nonetheless, these illnesses are not regarded diagnostic criteria for PCOS because not all obese women exhibit hyperandrogenism. Insulin resistance, which is present in the most obese and/or PCOS patients, is a risk factor for developing glucose intolerance and type 2 diabetes mellitus. Insulin resistance is higher and more severe in obese PCOS individuals than in non-obese PCOS patients.

The current review has summarized the influence of polymorphism in genes involved in steroidogenesis, gonadotropin action and control, insulin regulation that govern PCOS susceptibility and phenotypic heterogeneity. A candidate gene technique has been used in studies to give conclusive evidence for including or excluding any gene. Many genes are included in this article. However, only a handful have been proved to influence steroidogenesis pathways in PCOS women: CYP11A, CYP17, CYP19, 17HSD, SHBG, AR, RXR, KISS1, VDR. In addition, the genes LHCGR, INSR, FSHR, and GnRHR have been demonstrated to affect gonadotropin activity and control in PCOS women. Obesity and metabolic consequences are linked to the genes FTO, VEGF, ACE, and PPARG, revealing that obese PCOS patients had greater levels of Interleukin-1, PPARG, FTO, and VEGF when compared to control women. However, family investigations have revealed that PCOS has a genetic basis and that no single gene can fully explain the disease. Additionally, candidate gene approach has not provided conclusive results for any of the susceptible gene. As a result, the genetic markers studied thus far could aid in diagnosing the syndrome and its phenotypes, allowing for earlier involvement in co-morbidities and more personalized care.

## Data Availability

Not applicable.

## Abbreviations

PCOS: Polycystic ovary syndrome
USG: Ultrasonography
NIH: National Institute of Health
PCOM: Polycystic ovarian morphology
ESHRE: European Society of Human Reproduction and Embryology
ASRM: The American Society for Reproductive Medicine
AES: Androgen Excess Society
GnRH: Gonadotropin-releasing hormone
GnRHR: Gonadotropin-releasing hormone receptor
LH: Luteinizing hormone
LHCGR: Luteinizing hormone/choriogonadotropin receptor
FSH: Follicle stimulating hormone
FSHR: Follicle-stimulating hormone receptor
ACTH: Adrenocorticotrophic hormone
HSDs: Hydroxysteroid dehydrogenases
StAR: Steroidogenic acute regulatory protein
3-βHSD: 3β-hydroxysteroid dehydrogenase
17-βHSD: 17β-hydroxysteroid dehydrogenase
SHBG: Sex hormone-binding globulin
RSHBG: Receptor sex hormone-binding globulin
AR: Androgen receptor
TDT: Transmission disequilibrium test
INSR: Insulin receptor
GWAS: Genome-wide association studies
DENND1A: DENN Domain-Containing Protein 1A
THADA: Thyroid Adenoma Associated Protein
TOX3: Tox High Mobility Group Box Family Member 3
YAP1: Yes-Associated Protein 1
RAB5B: Ras-Related Protein Rab-5B
C9orf3: Chromosome 9 Open Reading Frame 3
HMGA2: High-Mobility Group AT-Hook 2
SUMO1P1/ZNF217: Pseudogene 1 / Zinc Finger Protein 217
HOMA-B: Homeostasis Model Assessment Of *β*-Cell Function
HAIR-AN: Hyperandrogenism, insulin resistance, and acanthosis nigricans
P450 scc: Side-chain cleavage enzyme
P450c17: 17-hydroxylase enzyme
P450arom: Aromatase enzyme
P450c21: 21-hydroxylase enzyme
NADPH: Nicotinamide Adenine Dinucleotide Phosphate
SNP: Single nucleotide polymorphism
FAI: Free Androgen Index
DHEAS: Dehydroepiandrosterone sulfate
E2: Estradiol
T: Testosterone
UTR: Untranslated Region
XCI: X- chromosome inactivation
BMP: Bone morphogenetic protein
BMI: Basal Metabolic Index
HPG: Hypothalamus-Pituitary-Gonadal
IL: Interleukin
ILRA: Interlekin-1 receptor antagonist
PPARG: Peroxisome proliferator activated receptor gamma
KISS: Kisspeptin
VDR: Vitamin-D receptor
RXR: Retinoid X receptor
FTO: Fat Mass and Obesity-Associated Protein
VEGF: Vascular Epithelial Growth Factor
ACE: Angiotensin converting enzyme
